# Microwave measurement of giant unilamellar vesicles in aqueous solution

**DOI:** 10.1038/s41598-017-18806-9

**Published:** 2018-01-11

**Authors:** Yan Cui, William F. Delaney, Taghi Darroudi, Pingshan Wang

**Affiliations:** 1 Department of Electrical and Computer Engineering, Clemson University, Clemson, SC 29634 USA; 2 Advanced Materials Research Laboratory, Clemson University, Anderson, SC 29625 USA

## Abstract

A microwave technique is demonstrated to measure floating giant unilamellar vesicle (GUV) membranes in a 25 *μ*m wide and 18.8 *μ*m high microfluidic channel. The measurement is conducted at 2.7 and 7.9 GHz, at which a split-ring resonator (SRR) operates at odd modes. A 500 nm wide and 100 *μ*m long SRR split gap is used to scan GUVs that are slightly larger than 25 *μ*m in diameter. The smaller fluidic channel induces flattened GUV membrane sections, which make close contact with the SRR gap surface. The used GUVs are synthesized with POPC (16:0–18:1 PC 1-palmitoyl-2-oleoyl-sn-glycero-3-phosphocholine), SM (16:0 Egg Sphingomyelin) and cholesterol at different molecular compositions. It is shown that SM and POPC bilayers have different dielectric permittivity values, which also change with measurement frequencies. The obtained membrane permittivity values, e.g. 73.64-*j*6.13 for POPC at 2.7 GHz, are more than 10 times larger than previously reported results. The discrepancy is likely due to the measurement of dielectric polarization parallel with, other than perpendicular to, the membrane surface. POPC and SM-rich GUV surface sections are also clearly identified. Further work is needed to verify the obtained large permittivity values and enable accurate analysis of membrane composition.

## Introduction

The dielectric property of a biomembrane is an important biophysical parameter^1^, which plays a critical role in various cellular physiological processes, including membrane ion permeation^2^, membrane potential formation^3^, and membrane surface hydration^4^. The property is also critical for molecular simulation and understanding^5,6^ of biological membrane organization^7^, dynamics and function^8,9^. It is shown that a small dielectric property change can lead to significantly different membrane behavior^10^. Furthermore, membrane dielectric property determines cell responses to external electric fields in dielectrophoresis^11^, impedance spectroscopy^12^ and electroporation^13^, which are important for scientific and technological development.

A commonly used technique for membrane dielectric property measurement is scanning probe microscopy (SPM), which includes electrostatic force microscopy^14–18^ and microwave microscopy^19^. The technique has nanoscale resolution, and is promising for investigating nanoscale dynamic membrane organizations, such as rafts^20^ and ultrathin bilayers^21^, and differentiating nanoparticle^22^. Nevertheless, SPM measurements are often conducted with supported lipid bilayers in air, and only recently in aqueous solution^14^. In force-based measurement, charges on membranes, among other factors, complicate data interpretation. The microwave microscopy needs cell fixation^23^ while its probe moves. The supporting substrate and the fixation process are likely to induce significant dielectric property changes in lipid bilayers and cell membranes. Other measurement methods include impedance spectroscopy^24^, environment-sensitive fluorescent microscopy^25^, electron paramagnetic resonance (EPR)^26^, imaging ellipsometry^27^, interferometry^28^, and dielectrophoretic spectroscopy^29^. These less commonly used methods may also need supported membranes or fixed cells. Measuring floating cells and model membranes, such as a giant unilamellar vesicle (GUV), in native or near native environment is still a challenge^30^. In this work, we demonstrate a microwave technique to spatially scan and quantify the dielectric property of floating GUV membranes in aqueous solution. We also show that the technique can clearly measure GUV membrane structures.

## Results

### Microwave scanning system

**Figure 1:**
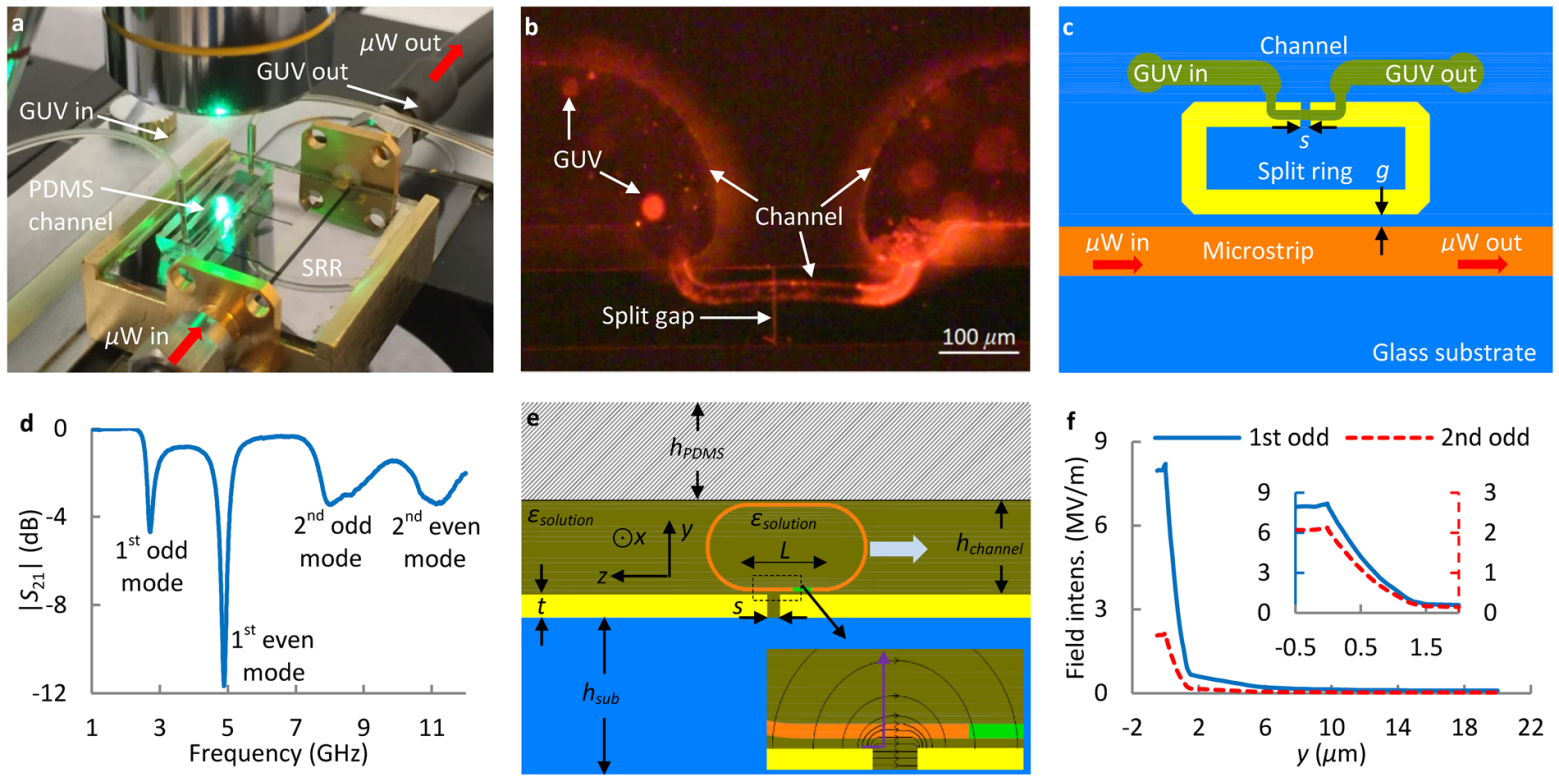
(**a**) A picture of the microwave (*µ*W) measurement setup, for which a vector network analyzer and a control computer are not shown. (**b**) A picture of GUVs in a PDMS microfluidic channel for microwave scanning by the split gap of an SRR. (**c**) A schematic of the SRR in (**b**). The planar SRR is built with conventional micro-/nano-fabrication processes. The dimension *g* for coupling is 3.5 *μ*m. The split gap is *s* = 500 nm wide and 100 *μ*m long. (**d**) Measured broadband |*S*_21_| with 0.1 M glucose-water solution in the channel. (**e**) A cross section schematic of a GUV under measurement by SRR split gap. The 18.8 *μ*m (*h*_*channel*_) channel is 25 *μ*m wide. The thicknesses of metal layer (*t*), glass substrate (*h*_*sub*_), and PDMS (*h*_*PDMS*_) are 0.54 *μ*m, 1 mm, and ~3 mm. The GUV flows in *z* direction. The zoom-in schematic illustrates electric field lines. (**f**) Simulated field distribution along *y* direction at the measurement gap center. The top metal surface is set as origin *y* = 0.

The key element of the scanning technique is the microwave device in Fig. 1a, for which the split gap of the split-ring resonator (SRR), Fig. 1b, determines 1-D scanning resolution. The SRR is coupled to a microstrip line as shown in Fig. 1c where the coupling strength is determined by factors like the gap dimension *g*. Analysis shows that only odd mode operation, from the transmission scattering parameter^31^ magnitude |*S*_21_| in Fig. 1d, provides high sensitivity measurements. At these frequencies, more than 10-times higher microwave electric fields are generated at the split gap, Fig. 1e, when compared with the fields in a conventional coplanar waveguide (CPW) structure carrying the same microwave probing power. At the SRR resonance frequencies, up to 50% of the microwave probing signal energy is coupled into the SRR. The field intensity variations in Fig. 1f show that (*i*) the microwave field is much stronger in and near the split gap than elsewhere. Besides, the field in the gap is parallel with GUV membrane and perpendicular to the field on the flat electrode surfaces, (*ii*) the top GUV membrane does not produce much signal due to very weak microwave fields, (*iii*) the vertical distributions of field intensity is different for 2.7 GHz (first odd mode) and 7.9 GHz (second odd mode).

GUV, a well-studied model membrane^32^ with well-characterized phase diagram^33,34^, is synthesized with different lipid molecule composition of POPC (16:0–18:1 PC 1-palmitoyl-2-oleoyl-sn-glycero-3-phosphocholine), SM (16:0 Egg Sphingomyelin) and cholesterol. To observe GUVs and their membrane domains while conducting microwave measurement, a fluorescent microscope (Fig. 1a) and two fluorescent labels, Rho-PE (2-dioleoyl-sn-glycero-3-phosphoethanolamine-N-(lissamine rhodamine B sulfonyl) (ammonium salt)) and DioC18 (3,3′-Dioctadecyloxacarbocyanine Percholorate) are used. The labels are for liquid-disordered phase *L*_*α*_ (POPC-rich) and liquid-ordered phase *L*_*o*_ (SM-rich) domains, respectively. Labels are reported to affect membrane structures^35,36^. But, measurements with label-free GUVs show that Rho-PE and DioC18 do not significantly affect microwave measurement results since they only account for ~1% of membrane lipid molecules. Additionally, the carrier medium and the solution inside the GUVs are from the same 0.1 M glucose-water solution bottle. Thus, the measured electrical signals can be attributed to GUV membrane bilayers and structures (Fig. 1e).

**Figure 2:**
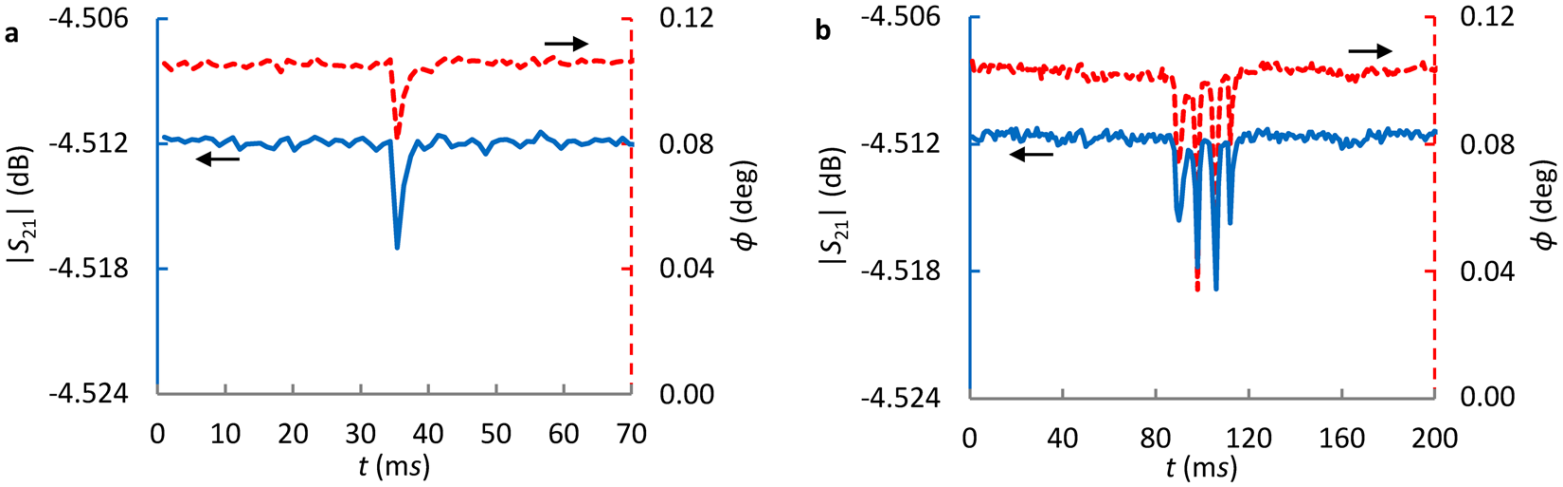
Measured |*S*_21_| and ∠*S*_21_ at 2.7 GHz of (**a**) a ~17.4 *μ*m and (**b**) a ~31 *µ*m GUV synthesized with 0/100/0 of POPC/SM/Cholesterol. Angle *ϕ* = ∠*S*_21_-103.9° is plotted for ∠*S*_21_ for better presentation.

For a spherical GUV with diameter smaller than the channel height, it is difficult to control its vertical position for measurement, Fig. 1e. As a result, measured signals, as shown in Fig. 2a, may vary significantly even for identical GUVs. Furthermore, a spherical GUV makes data interpretation and corresponding dielectric property extraction complicated. Thus, a flat GUV membrane, as illustrated in Fig. 1e, is targeted in this work. For large GUVs, membrane folding may happen probably when different GUV sections experience different velocities induced by factors like local friction force variations along the channel. Figure 2b shows the measured signal of a ~31 *μ*m SM GUV. For even larger GUVs, they tend to break into pieces when entering the smaller channel section. Therefore, we focus on GUVs that are slightly larger than 25 *μ*m in diameter. It is estimated that a 25 *μ*m GUV will yield a flattened section with *L* ≈ 11 *μ*m (*z* direction) which is ~20.5 *µ*m wide in *x* direction (Fig. 1e) inside the 18.8 *μ*m × 25 *μ*m channel. The estimation is consistent with the observations in ref.^37^ even though the GUVs are restricted in two directions instead of one. As a result, the bottom flat membrane will be scanned.

### Microwave responses of SM and POPC GUV membranes

**Figure 3:**
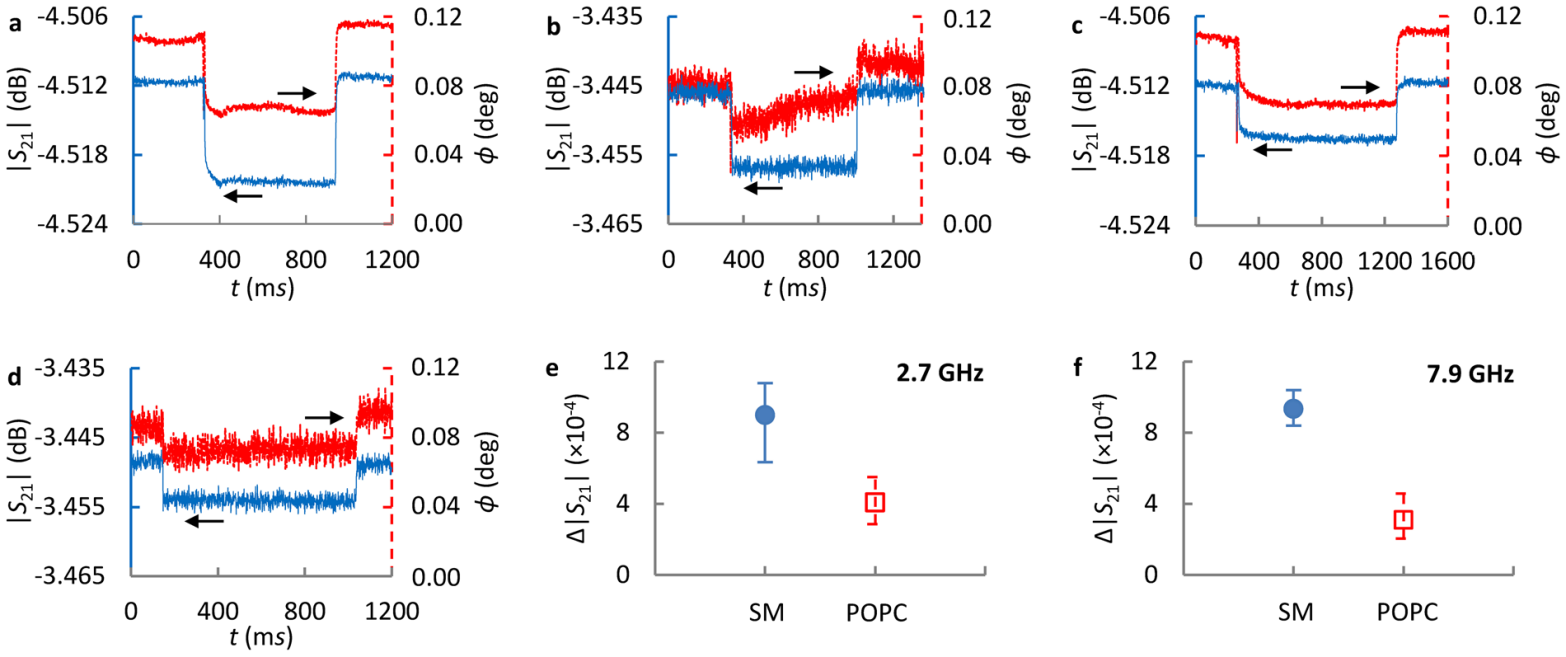
Measured |*S*_21_| and ∠*S*_21_ of single GUV membranes. (**a**) A GUV of 100% SM at 2.7 GHz, and (**b**) 7.9 GHz. (**c**) A POPC GUV measured at 2.7 GHz and (**d**) 7.9 GHz. Angle *ϕ* = ∠*S*_21_-*θ*_0_ (*θ*_0_ = 103.9° for (**a**) and (**c**), and 339.88° for (**b**) and (**d**)) is plotted for ∠*S*_21_ for better presentation. (**e**) The measured |*S*_21_| change, i.e. Δ|*S*_21_|, induced by SM and POPC GUV membranes at 2.7 GHz, and (**f**) at 7.9 GHz. The solid circles and open squares indicate average values *μ* calculated from 10 GUVs. The positive and negative error values are calculated by Δ|*S*_21_|_max_-*μ* and *μ*-Δ|*S*_21_|_min_, respectively.

Figure 3a–f show the measured *S*_21_ of single GUV membranes with 100% SM and 100% POPC, respectively, and at 2.7 and 7.9 GHz. It takes ~0.6 *s* to 0.9 *s* to scan the GUVs with the 500 nm split gap. The relatively long time indicates slow GUV translocation through the channel, unlike the smaller or larger GUV in Fig. 2. The slightly larger noise at 7.9 GHz is likely from measurement system. Nevertheless, the results show well behaved and clearly differentiable GUV membrane signals. As expected, the flat signal level indicates flat GUV membranes, Fig. 1e.

Figure 3a–d indicate that SM GUVs induce larger *S*_21_ changes at both frequencies. Nevertheless, the variations of GUV size and shape could also induce the observed differences since visual estimation of GUV size is not accurate. Additionally, the fluidic channel causes GUV deformation, which could induce different dielectric property changes for SM and POPC GUVs. To address the uncertainties, 10 GUVs from the same synthesis batch are measured individually to obtain the average values, *µ*, and corresponding standard deviation, *σ*, as illustrated in Fig. 3e and f. The obtained (*µ*, σ) of Δ|*S*_21_| are (8.99 × 10^−4^, 1.56 × 10^−4^) at 2.7 GHz, (9.35 × 10^−4^, 0.57 × 10^−4^) at 7.9 GHz for SM, and (4.09 × 10^−4^, 0.87 × 10^−4^) at 2.7 GHz, (3.11 × 10^−4^, 0.97 × 10^−4^) at 7.9 GHz for POPC. These results indicate that both SM and POPC GUV membranes induce significant Δ|*S*_21_| while there are also substantial Δ|*S*_21_| variations. The variations may come from the uncertainties discussed above. Secondly, Δ|*S*_21_| is clearly different for SM and POPC lipids, probably due to longer SM lipid molecules and corresponding thicker membrane^38^.

### GUV membrane domains

**Figure 4:**
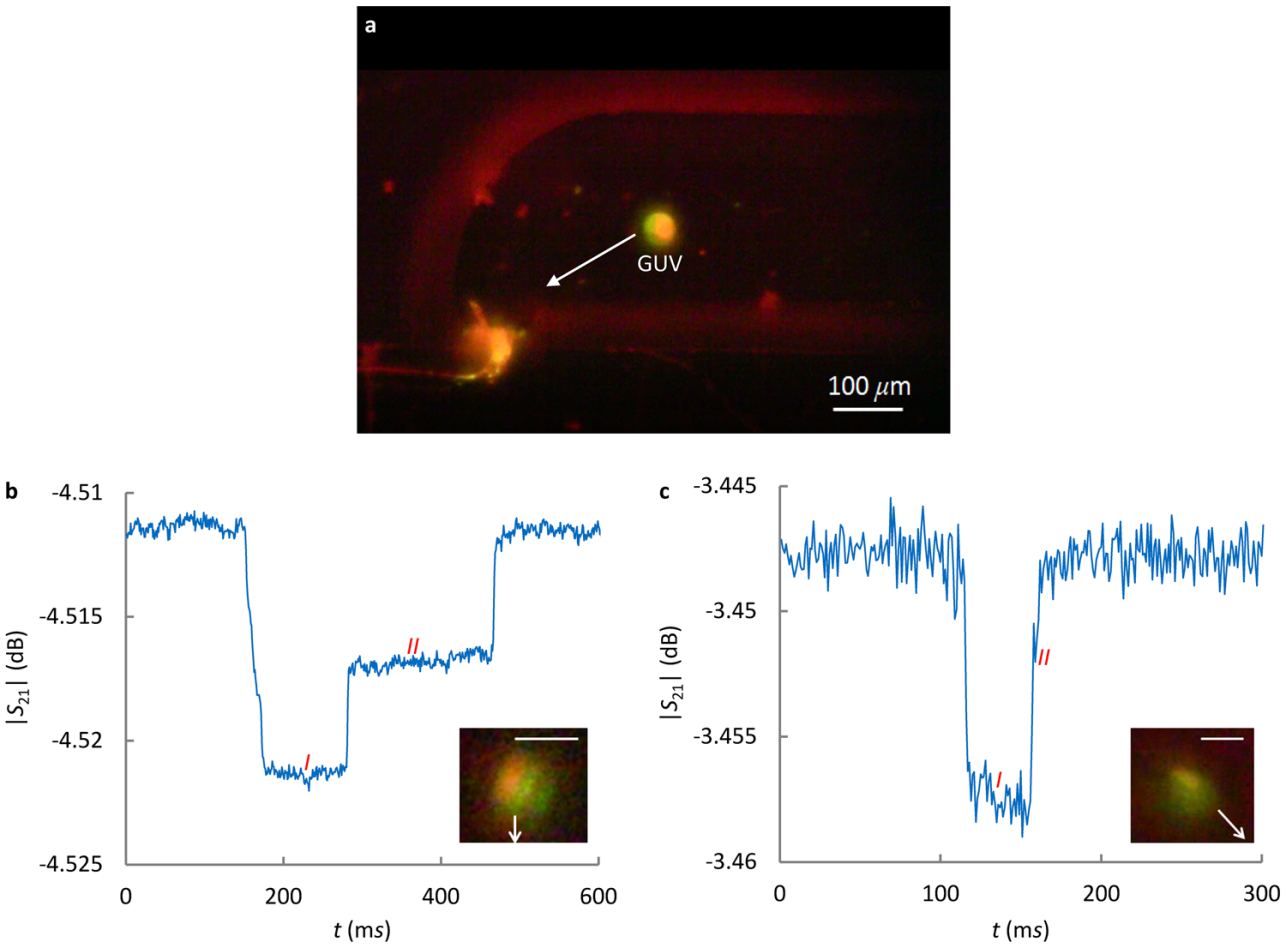
(**a**) A fluorescent picture of a GUV with SM and POPC lipids (synthesized with SM/POPC/Cholesterol ratio of 65/10/25). Measured |*S*_21_| of a single GUV at (**b**) 2.7 GHz and (**c**) 7.9 GHz. The arrows in the zoom-in pictures indicate the flow directions before these GUVs enter the narrow channel. Scale bar: 25 *μ*m.

For GUVs synthesized with SM, POPC and cholesterol mixtures, liquid-disordered phase *L*_*α*_ (POPC-rich) and liquid-ordered phase *L*_*o*_ (SM-rich) domains can be formed, such as that shown in Fig. 4a. In experiment, it is observed that the raft-like domain (green color) always locate at the front of a GUV in the microfluidic channel, probably caused by the moving outer fluid^37,39^. When a flattened GUV slowly passes over the 500 nm split gap, the raft-like domain appears first, and induces the first Δ|*S*_21_|, i.e. Section *I* in Fig. 4b, followed by the second Δ|*S*_21_|, i.e. Section *II* in Fig. 4b, which indicates the passage of the non-raft domain over the split gap.

Table 1 summarizes the measurement results, three at 2.7 GHz and one at 7.9 GHz. All 2.7 GHz measurements show clear Sections *I* and *II*. The Section *II* of 7.9 GHz measurement (Fig. 4c) is less clearer, probably due to small POPC domains, as indicated in the zoom-in picture. The average |*S*_21_| changes (∆|*S*_21_|) in Table 1 are obtained by calculating |Average(*S*_21,no GUV_) − Average(*S*_21,Section *I*,*II*_)| in Fig. 4. It is shown that *S*ection *I* is SM-rich, and Section *II* is POPC-rich. This also agrees with the fluorescent label indications. Therefore, the proposed measurement can detect different domains on a GUV. It is worth noting that the addition of cholesterol does not significantly change Δ|*S*_21_| for the SM response. A possible reason is that the dipole moment of cholesterol molecule is one order of magnitude smaller than that of SM and POPC lipid molecule, as pointed out in ref.^8^.

**Table 1:** Average Δ|*S*_21_| induced by Section *I* and *II*.

Section#	Label color	Measurement frequency	Δ|*S*_21_|(×10^−4^)	Main component*
*I*	Green	2.7 GHz	10.64	SM
		2.7 GHz	7.32	SM
		2.7 GHz	9.29	SM
		7.9 GHz	7.91	SM
*II*	Orange	2.7 GHz	4.55	POPC
		2.7 GHz	2.92	POPC
		2.7 GHz	4.74	POPC
		7.9 GHz	2.71	POPC

*The main lipid components are determined by comparing their Δ|*S*_21_| values with those in Fig. 3e and f.

### GUV membrane permittivity

**Figure 5:**
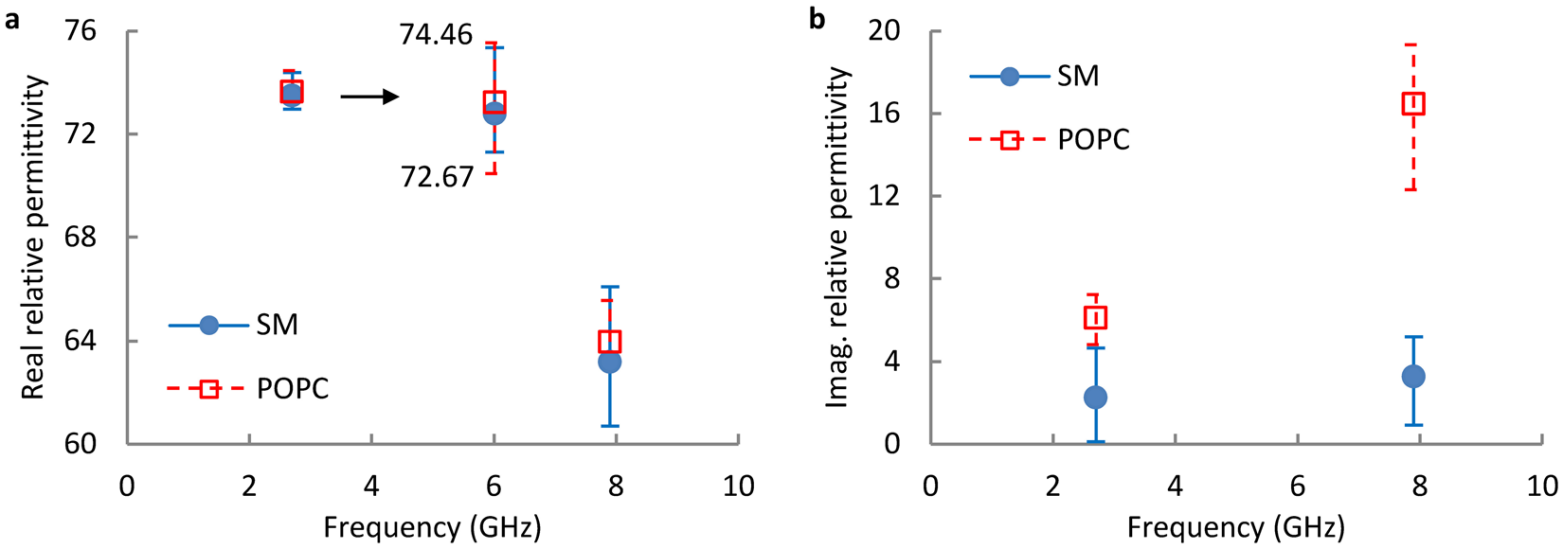
The obtained (**a**) real and (**b**) imaginary relative permittivity of SM and POPC GUVs at 2.7 GHz and 7.9 GHz.

The quantitative dielectric properties of GUV membranes can be obtained from the measured scattering parameters provided that appropriate equivalent circuit models of the measurement system are established and calibrated. Such a model is presented in Supporting Information together with data process procedures. The obtained dielectric properties of those measured GUVs in Fig. 3 are presented in Fig. 5. It is shown that POPC and SM have similar real permittivity values (*ε*′, Fig. 5a) but significantly different imaginary components (*ε*″, Fig. 5b) at both frequencies, which indicates that POPC absorbs more microwave than SM lipid.

The obtained GUV membrane permittivity values in Fig. 5a are more than 10 times higher than that of the supported bilayers in air^15^ and electrolyte^14^ despite the fact that the lipid molecules used in these experiments are different and electrolyte reduces hydration water permittivity^40^. A possible reason is that the measured permittivity values in Fig. 5 are from lipid polarizations perpendicular to those in previous efforts. As shown in Fig. 1e, the fields between the 500-nm wide split gap, *E*_*||*_, is parallel with the membrane surface plane while the fringing fields near the gap edge, *E*_*⊥*_, is perpendicular to GUV membrane. But *E*_*||*_ is much stronger than *E*_*⊥*_ and account for more than 90% of the electric field energy for GUV measurement. Thus, the lipid polarization process for Fig. 5 is perpendicular to the process in SPM efforts, i.e. Figure 5 is for *ε*_*||*_ rather than *ε*_*⊥*_. It is predicted that *ε*_*||*_ values are very high^41^, much higher than *ε*_*⊥*_, albeit the results are calculated at DC, not at GHz frequencies. Regardless, the scanning technique in Fig. 1 provides a method to measure the permittivity values (*ε*_*||*_′ and *ε*_*||*_″) that are difficult to access by any of current techniques. A second possible reason is data extraction uncertainty. To obtain GUV membrane permittivity values in Fig. 5, GUV membranes are assumed to be in contact with the smooth and flat electrode surfaces around the SRR split gap, Fig. 1e. This assumption is questionable because there will be thin layers of liquids on electrode-surface and GUV membrane outer surface, e.g. hydration water on GUV membrane. As a result, GUV membrane will be elevated above electrode surface by the liquid thin layers. It is estimated that a 100-nm elevation will result in a factor of 0.107 reduction of probing field intensity, as shown in the zoom-in illustration of Fig. 1f. As expected, the obtained GUV permittivity values (expectation *μ*) are lower, 73.17-*j*1.34 at 2.7 GHz, 62.75-*j*0.65 at 7.9 GHz for SM, and 73.34-*j*5.66 at 2.7 GHz, 63.61-*j*15.41 at 7.9 GHz for POPC. But the effects are not significant even though the exact GUV elevation height is still uncertain. Further complicating accurate data extraction are the uncertainties associated with the permittivity values of surface water and confined water, which are a subject of active investigation^42–45^. Besides, the developed equivalent circuit model may not accurately consider factors like stray effects. Thus, studying model systems of known dielectric constants and independent verification of *ε*_||_ measurements are needed in future investigations.

## Discussion

The spatial resolution of the 1-D scanning in Fig. 1 is determined by the ~500 nm split gap. Engineering the split gap dimensions in both *x* and *y* directions, a high resolution 2-D scanning can be achieved provided that measurement sensitivity is sufficient. The scanning technique is also intrinsically label-free even though fluorescent labels are used in this work for better visual guidance. The label-free characteristic is beneficial for various investigations since labels are known to affect membrane structures and functions^35,36^. Furthermore, the number of microwave frequency points can be increased for broadband dielectric spectroscopy studies of GUV membrane dynamics^46–50^. The technique complements fluorescence microscopy and interferometric scattering microscopy which are the currently available methods for lipid-domain investigations with millisecond time resolutions^51^. Additionally, it is shown that well characterized broadband dielectric properties enable label-free determination of molecular compositions in liquids^52^. Thus, the scanning technique could be further developed for GUV membrane molecular composition analysis in a label-free manner. Currently, there is a lack of such methods.

In summary, the microwave technique provides high sensitivity scanning of GUV membrane dielectric properties. It is shown that floating SM and POPC lipid bilayers have significantly different dielectric properties, mainly in microwave absorptions. The properties also depend on measurement frequencies. Thus, GUV domains, which are mainly formed with different lipid molecules, are clearly identified from microwave scanning. The obtained GUV dielectric property values, based on algorithms developed for the scanning technique, are much larger than those obtained from other techniques. Nevertheless, the permittivity is from molecular polarizations parallel to GUV surface. Currently, there is no other technique that can measure such properties. Further investigation is needed to clarify the accuracy of the obtained permittivity values, increase scanning resolutions, analyze membrane dynamics, and determine membrane molecular compositions.

## Methods

### GUV electroformation synthesis

The GUV synthesis uses an electroformation process in ref.^37^ with modifications. Prior to GUV preparation, 10 mg/mL Chol (Cholesterol, purchased from Sigma-Aldrich, St. Louis, MO, USA), 10 mg/mL SM (16:0 Egg Sphingomyelin, purchased from Avanti Lipids Polar, Alabaster, AL, USA) and POPC (16:0–18:1 PC 1-palmitoyl-2-oleoyl-sn-glycero-3-phosphocholine, purchased from Avanti Lipids Polar, Alabaster, AL, USA), and 1 mg/mL DioC18 (3,3′-Dioctadecyloxacarbocyanine Percholorate, purchased from Sigma-Aldrich, St. Louis, MO, USA) and 1 mg/mL Rho-PE (2-dioleoyl-sn-glycero-3-phosphoethanolamine-N-(lissamine rhodamine B sulfonyl) (ammonium salt), purchased from Avanti Lipids Polar, Alabaster, AL, USA) in chloroform are taken out of a −20 °C freezer and naturally warmed to room temperature. Two indium tin oxide (ITO) glass slides are cleaned by ethanol and heated on a 55 °C hotplate. Two glass test tubes are 3/4 filled with chloroform: **A** for washing syringes and **B** for sample preparation. Then draw 120 *μ*L chloroform from test tube **B** and drop it into a clean test tube **C**. For 100% SM or POPC GUV synthesis, 35 *μ*L SM- or POPC-chloroform solution at room temperature is dropped into a tube **C**, and 1 *μ*L DioC18 (for SM) or Rho-PE (for POPC) is added; for 65/10/25 SM/POPC/Chol GUV synthesis, 25.4/4.23/5.37 *μ*L SM-, POPC-, and Chol- chloroform solutions at room temperature are dropped into a tube **C** with 1 *μ*L DioC18 and 1 *μ*L Rho-PE. The next step is to draw 80 *μ*L Chol/lipids/labels mixture chloroform solution from the tube **C** and drop the solution on the ITO coating surface of a heated ITO glass slide, followed by immediate and uniform spreading of the solution over the top 1/3 of the slide. The two ITO glass slides are then vacuum-desiccated at ~65 Torr for 2 hours. Draw 500 *μ*L glucose-water solution at 0.1 M concentration, which is prepared in a conical tube **D**, and drop the solution into an O-ring rubber area. The O-ring rubber is placed on the glass slide to circle a lipid film area. The area is then sealed with the other vacuum-desiccated ITO glass slide and to form a chamber. The top and bottom surfaces of the chamber should be ITO coating surfaces. For electroswelling, the rubber-glass chamber is placed into an incubator at a temperature of ~60 °C. A 1 V 10 Hz sinusoidal voltage is applied to the ITO electrodes. Lipid bilayers will spontaneously vesiculate in 2 hours, and then collected in a tube **E** with 3 mL 0.1 M glucose-water solution, which is from the same conical tube **D** ^53^.

Following the above recipe, ~5% GUVs have slightly larger than 25 *μ*m^37^ diameters for our experiments, which are conducted at room temperatures, lower than the miscibility transition temperature (~40 °C) of SM/POPC/Chol: 65/10/25^34^. The use of fluorescent labels only slightly affects this temperature^54^. So the GUVs synthesized with the above ternary ratio are effectively phase separated into two different regions^34^.

### Flow system and data collection

An NE-1000 Programmable Single Syringe Pump (New Era Pump Systems Inc., Farmingdale, NY, USA) is used to push GUVs into and out of the channel. A 2 *μ*L/min flow rate is set in all experiments. A vector network analyzer (VNA) for *S*-parameter measurement is connected to a laptop and operated with LabView and graphical codes (National Instruments Corporation, Austin, TX, USA). The measured real and imaginary parts of *S*_21_ are saved with LabView codes. A 1 kHz intermediate frequency bandwidth (IFBW) is used for VNA measurement.

## Electronic supplementary material

Supplementary Information
