# Supplementary material for: Microwave measurement of giant unilamellar vesicles in aqueous solution
Source: Sci Rep. 2018 Jan 11;8:497. doi: 10.1038/s41598-017-18806-9 (PMC5764977; doi:10.1038/s41598-017-18806-9)
Supplement: Supplementary file 1 — Supplementary Information [file 41598_2017_18806_MOESM1_ESM.pdf]

Microwave measurement of giant unilamellar vesicles in aqueous solution

Yan Cui[1], William F. Delaney[1], Taghi Darroudi[2] & Pingshan Wang[1]

[1]Department of Electrical and Computer Engineering, Clemson University, Clemson, SC 29634, USA. [2]Advanced Materials Research Laboratory, Clemson University, Anderson, SC 29625, USA. Correspondence and requests for materials should be addressed to P. W. (email: pwang@clemson.edu).

Supplementary information

Analytical model of the SRR and complex permittivity extraction of GUV membranes

To analyze, design, and extract the permittivity of MUT locating at the split, an appropriate mathematic model of the proposed SRR is required. Some equivalent circuit-based models have been proposed to meet the demand [1]-[3]. However, these existing models have to fit and adjust lumped-element parameters to agree with the measured *S*-parameters. Moreover, these fitted parameters are not always unique, which brings a great uncertainty for permittivity extraction. To address this issue, a novel analytical model is proposed to obtain *S*-parameters of the SRR based on necessary structural dimensions and dielectric information.

The proposed SRR can be modeled as shown in Fig. S1, which is composed of a coupled line and a gaped ML, discussed in Section 1 and 2, respectively. Then, using the even-odd mode analysis technique [4], they are combined together for the final 2-port *S*-parameters in Section 3. For the well-matched MLs on the left and right ends, they only shift the phase of the combined *S*-parameters. So the total phase of the SRR will be revised using the measurement result as reference.

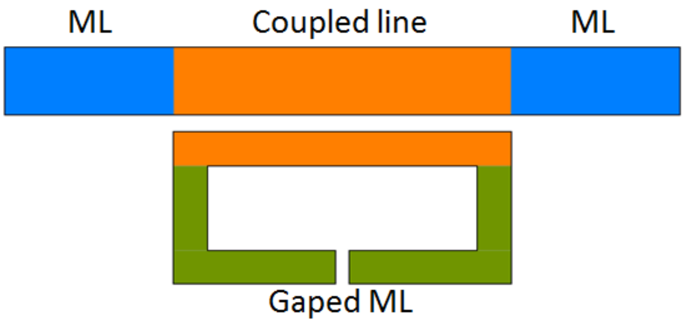

Fig. S1 Decomposition of the SRR. Blue: ML; orange: coupled line; green: gaped ML.

1 *S*-parameters of Asymmetric Coupled Line

For a four-port asymmetric coupled line made up by two adjacent MLs *a* and *b* with a spacing *s* and arbitrary widths W_1 and W_2 in Fig. S2, its *S*-parameters can be expressed as

$$[S]=\begin{bmatrix} S_{11} & S_{12} & S_{13} & S_{14} \\ S_{21} & S_{22} & S_{23} & S_{24} \\ S_{31} & S_{32} & S_{33} & S_{34} \\ S_{41} & S_{42} & S_{43} & S_{44} \end{bmatrix}=\begin{bmatrix} S_{11} & S_{21} & S_{31} & S_{41} \\ S_{21} & S_{11} & S_{41} & S_{31} \\ S_{31} & S_{41} & S_{33} & S_{43} \\ S_{41} & S_{31} & S_{43} & S_{44} \end{bmatrix} \quad (1)$$

where S_{11}=S_{22}, S_{21}=S_{12}, S_{33}=S_{44}, S_{43}=S_{34}, S_{31}=S_{13}=S_{42}=S_{24}, S_{41}=S_{14}=S_{32}=S_{23}, because of the symmetry and reciprocity [5].

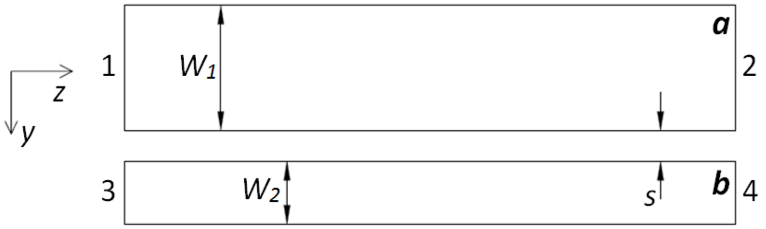

Fig. S2 Four-port asymmetric coupled line with a spacing *s* and arbitrary widths W_1 and W_2.

Under the assumption of transverse electromagnetic (TEM) propagation, the wave propagation along *y* direction in Fig. S2, is described by conventional transmission-line theory [6] as

$$\frac{d[V]}{dy}=-[z][I] \quad (2)$$

where $[V]=\begin{bmatrix} V_1 \\ V_3 \end{bmatrix}$, $[I]=\begin{bmatrix} I_1 \\ I_3 \end{bmatrix}$, the elements V_i and I_i (i=1, 3) represent the voltage and current at the port *i* of the network. And the impedance matrix is

$$[z]=\begin{bmatrix} z_{11} & z_{13} \\ z_{31} & z_{33} \end{bmatrix}=\begin{bmatrix} z_{11} & z_{31} \\ z_{31} & z_{33} \end{bmatrix} \quad (3)$$

where z_{13}=z_{31} because of the reciprocity [5]. Now we solve the following eigenvalue equation for [z]:

$$([z]-\lambda[E])[x]=0 \tag{4}$$

where [E] is the unity matrix, and [x] corresponding to voltage modes is eigenvectors of [z], solved as

$$\overrightarrow{x_a}=\begin{bmatrix} m \\ 1 \end{bmatrix}=\begin{bmatrix} \dfrac{z_{11}+z_{33}-\sqrt{z_{11}^2-2z_{11}z_{33}+4z_{31}^2+z_{33}^2}}{2z_{31}}-\dfrac{z_{33}}{z_{31}} \\ 1 \end{bmatrix} \tag{5}$$

$$\overrightarrow{x_b}=\begin{bmatrix} n \\ 1 \end{bmatrix}=\begin{bmatrix} \dfrac{z_{11}+z_{33}+\sqrt{z_{11}^2-2z_{11}z_{33}+4z_{31}^2+z_{33}^2}}{2z_{31}}-\dfrac{z_{33}}{z_{31}} \\ 1 \end{bmatrix} \tag{6}$$

The eigenvalue [λ] is also calculated as

$$\lambda_1=\frac{z_{11}+z_{33}-\sqrt{z_{11}^2-2z_{11}z_{33}+4z_{31}^2+z_{33}^2}}{2} \tag{7}$$

$$\lambda_3=\frac{z_{11}+z_{33}+\sqrt{z_{11}^2-2z_{11}z_{33}+4z_{31}^2+z_{33}^2}}{2} \tag{8}$$

Then, from the capacitance matrix $\begin{bmatrix} Q_1 \\ Q_3 \end{bmatrix}=\begin{bmatrix} c_{11} & c_{13} \\ c_{31} & c_{33} \end{bmatrix}\begin{bmatrix} V_1 \\ V_3 \end{bmatrix}=\begin{bmatrix} c_{11} & c_{31} \\ c_{31} & c_{33} \end{bmatrix}\begin{bmatrix} V_1 \\ V_3 \end{bmatrix}$, we have the following static capacitances to ground per unit length for each conductor (a and b):

(1) Differential Mode (π)

$$C_{a\pi}=c_{11}+\frac{1}{m}c_{31} \tag{9}$$

$$C_{b\pi}=mc_{31}+c_{33} \tag{10}$$

(2) Common Mode (c)

$$C_{ac}=c_{11}+\frac{1}{n}c_{31} \tag{11}$$

$$C_{bc}=nc_{31}+c_{33} \tag{12}$$

where the self-capacitance coefficients c_{11}, c_{33} are solved using c_p+2c_f from eqs. (3)-(4) in [7], the mutual-capacitance coefficients c_{31} for two adjacent asymmetric lines is solved using C_0^{ACPS}•ε_{eff}^{ACPS} from eqs. (10) and (39) in [8].

To order to obtain the same modal impedances for two conductors, in the above equations, $C_{a\pi}=C_{b\pi}$ and $C_{ac}=C_{bc}$ should be met. Then we have

$$m = \frac{-(c_{33}-c_{11}) - \sqrt{(c_{33}-c_{11})^2 + 4{c_{31}}^2}}{2c_{31}} \tag{13}$$

$$n = \frac{-(c_{33}-c_{11}) + \sqrt{(c_{33}-c_{11})^2 + 4{c_{31}}^2}}{2c_{31}} \tag{14}$$

It is worth noting that mn=-1, which will be used in some later calculations. For a special case when the conductor a and b in Fig. S2 are the same, m=-1, n=1. Then, the mode impedances are written as

$$z_{a\pi} = \frac{1}{v_{a\pi}C_{a\pi}} = \frac{1}{v_{a\pi}(c_{11}+c_{31}/m)} \tag{15}$$

$$z_{b\pi} = \frac{1}{v_{b\pi}C_{b\pi}} = \frac{1}{v_{b\pi}(mc_{31}+c_{33})} \tag{16}$$

$$z_{ac} = \frac{1}{v_{ac}C_{ac}} = \frac{1}{v_{ac}(c_{11}+c_{31}/n)} \tag{17}$$

$$z_{bc} = \frac{1}{v_{bc}C_{bc}} = \frac{1}{v_{bc}(nc_{31}+c_{33})} \tag{18}$$

Because of $C_{a\pi}=C_{b\pi}$ and $C_{ac}=C_{bc}$, we have

$$Z_{\pi} = \frac{1}{v_{\pi}(c_{11}+c_{31}/m)} \tag{19}$$

$$Z_{c} = \frac{1}{v_{c}(c_{11}+c_{31}/n)} \tag{20}$$

The reflection and transmission coefficients of each mode in terms of corresponding mode impedances and electrical lengths are expressed as

$$\Gamma_x = j[[\frac{Z_x}{Z_0} - \frac{Z_0}{Z_x}]/\sin\theta_x]/\phi_x \tag{21}$$

$$T_x = 2/\phi_x \tag{22}$$

$$\phi_x = 2\cos\theta_x + j(\frac{Z_x}{Z_0} + \frac{Z_0}{Z_x})/\sin\theta_x \tag{23}$$

For the asymmetric a and b (as a result, we use π and c instead of odd and even denoting modes), these equations (21)-(23) have verified by V. K. Tripathi *et al* [9].

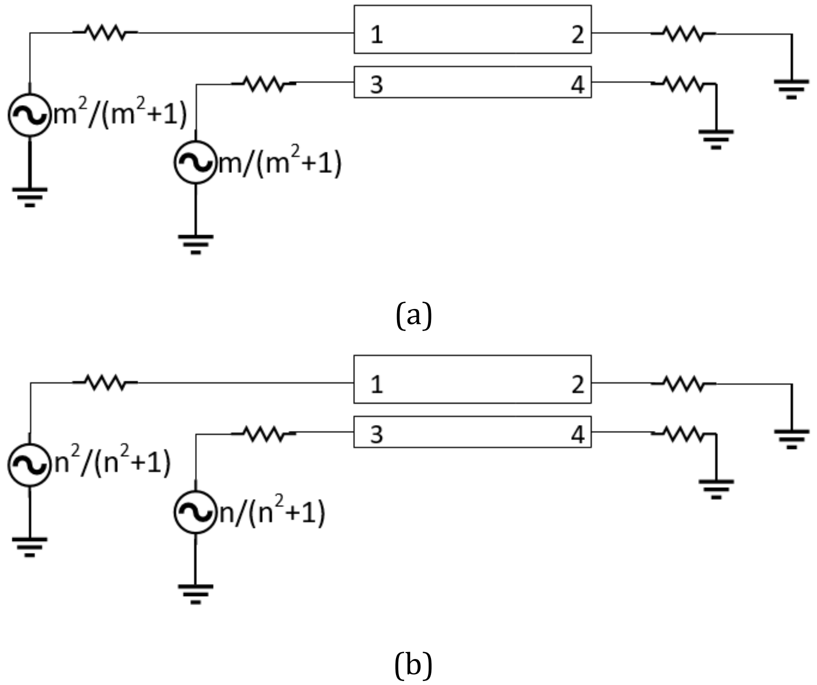

Fig. S3 System of generators for derivation of the S-parameters for an overall 1 V input signal at Port 1: (a) π Mode; (b) c Mode.

S-parameters of this system can be solved using voltage generators at each matched port, e.g., S_{11}, S_{21}, S_{31} and S_{41} can be determined by the emerging voltage waves at all ports to an incident voltage wave at Port 1, shown in Fig. S3.

For an applied 1 V voltage at Port 1, the voltages at two conductors can be solved as

(1) Differential Mode (π)

$$\frac{1}{1+(\frac{1}{m})^2} = \frac{m^2}{m^2+1} \text{ for Port 1} \tag{24}$$

$$\frac{\frac{1}{m}}{1+(\frac{1}{m})^2} = \frac{m}{m^2+1} \text{ for Port 3} \tag{25}$$

(2) Common Mode (c)

$$\frac{1}{1+(\frac{1}{n})^2}=\frac{n^2}{n^2+1} \quad \text{for Port 1} \tag{26}$$

$$\frac{\frac{1}{n}}{1+(\frac{1}{n})^2}=\frac{n}{n^2+1} \quad \text{for Port 3} \tag{27}$$

One can easily prove that

$$\frac{m^2}{m^2+1}+\frac{n^2}{n^2+1}=1 \tag{28}$$

and $$\frac{m}{m^2+1}+\frac{n}{n^2+1}=0 \tag{29}$$

The reflection and transmission coefficients for each mode are the same on each of the conductor *a* and *b* since $z_{a\pi}=z_{b\pi}$ and $z_{ac}=z_{bc}$. Now we obtain

$$S_{11}=\frac{m^2}{m^2+1}\Gamma_{OE}+\frac{n^2}{n^2+1}\Gamma_{EE} \tag{30}$$

$$S_{21}=\frac{m^2}{m^2+1}T_{OE}+\frac{n^2}{n^2+1}T_{EE} \tag{31}$$

$$S_{31}=\frac{m}{m^2+1}\Gamma_{OE}+\frac{n}{n^2+1}\Gamma_{EE} \tag{32}$$

$$S_{41}=\frac{m}{m^2+1}T_{OE}+\frac{n}{n^2+1}T_{EE} \tag{33}$$

Similarly, to apply an overall 1 V voltage at Port 3, the distribution of voltages at two conductors shown in Fig. S4 can be solved:

(1) Differential Mode (π)

$$\frac{m}{m^2+1} \quad \text{for Port 1} \tag{34}$$

$$\frac{1}{m^2+1} \quad \text{for Port 3} \tag{35}$$

(2) Common Mode (c)

$$\frac{n}{n^2+1} \text{ for Port 1} \tag{36}$$

$$\frac{1}{n^2+1} \text{ for Port 3} \tag{37}$$

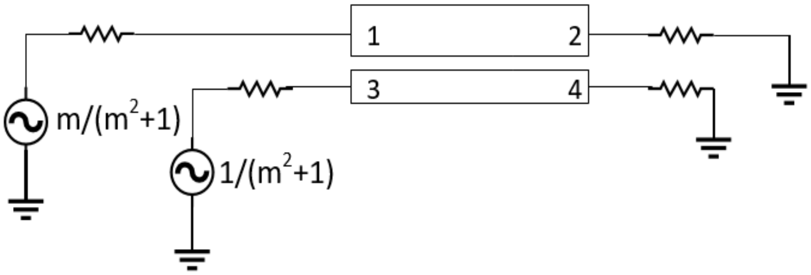

(a)

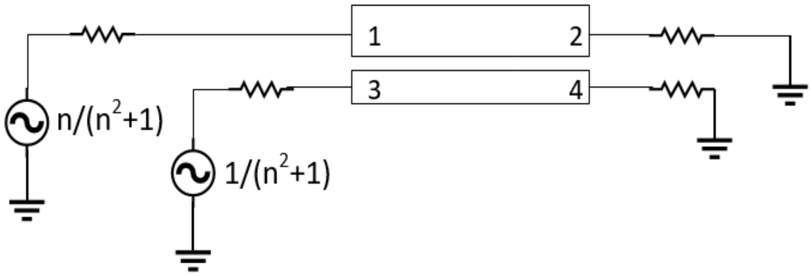

(b)

Fig. S4 System of generators for derivation of the S-parameters for an overall 1 V input signal at port 3: (a) π Mode; (b) c Mode.

Similarly, S_{13}, S_{33}, S_{43}, S_{23} can be also solved:

$$S_{13} = \frac{m}{m^2+1}\Gamma_{OE} + \frac{n}{n^2+1}\Gamma_{EE} \tag{38}$$

$$S_{33} = \frac{1}{m^2+1}\Gamma_{OE} + \frac{1}{n^2+1}\Gamma_{EE} \tag{39}$$

$$S_{43} = \frac{1}{m^2+1}T_{OE} + \frac{1}{n^2+1}T_{EE} \tag{40}$$

$$S_{23} = \frac{m}{m^2+1}T_{OE} + \frac{n}{n^2+1}T_{EE} \tag{41}$$

Let us go back to the matrix (1), we can find that all remaining S-parameters are also obtained therefore from S_{11}=S_{22}, S_{21}=S_{12}, S_{33}=S_{44}, S_{43}=S_{34}, S_{31}=S_{13}=S_{42}=S_{24}, S_{41}=S_{14}=S_{32}=S_{23}.

2 Reflection Coefficient of Gaped ML

Some publications have presented the modeling of the split area. The analytic mathematic expression is very complicated even for the simplest case that only includes necessary conductor and dielectric substrate [10]-[12]. These models do not consider a top dielectric layer above the metal, e.g., the microfluidic channel filled with a solution. In this section, a π-network of capacitors [13] shown in Fig. S5 will be used to model this area for an effective and simple quantification.

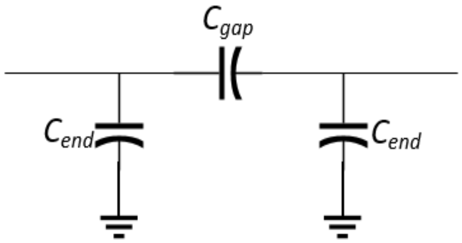

Fig. S5 π-network of capacitors for the equivalent circuit of the split.

The following will introduce how to solve C_{end} and C_{gap} in Fig. S5, respectively. Although some fitting equations are reported [13], these equations are generally not applicable to the multiple layer system of substrate/metal layer/GUV bottom surface/solution inside the GUV/GUV top surface/PDMS/air shown in Fig. S6. So, some more practical and applicable to the multiple layer system for C_{gap} calculation needs to be established. For C_{end}, however, most of electric fields emitted from edge only penetrate substrate material and terminate at the back conductor, rather than another conductor, so the fitting equation of C_{end} is applicable. According to [13],

$$C_{end} = 0.5\{12W(\frac{\varepsilon_{sub}}{9.6})^{0.9}(\frac{s}{W})^{0.8675}\exp(2.043(\frac{W}{h})^{0.12})\}(pF) \quad (42)$$

where s is the gap width, ε_{sub} and h are the dielectric constant and thickness of the substrate, respectively, and W is the width of the ML, which is different from the length w and perpendicular to the cross section in Fig. S6.

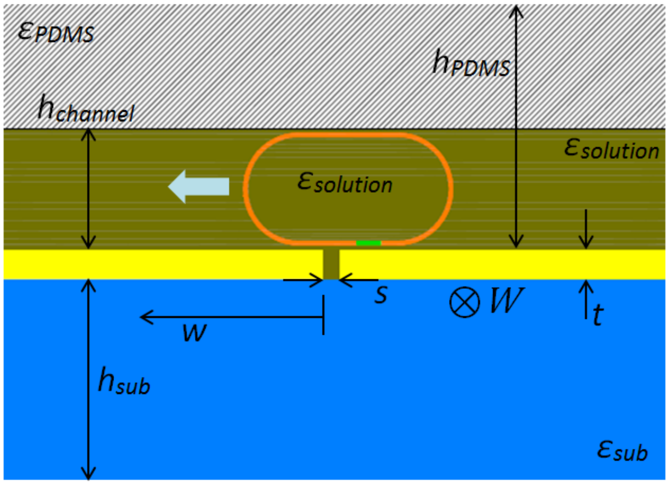

Fig. S6 Cross-section of the substrate/metal layer/GUV bottom surface/liquid inside the GUV/GUV top surface/PDMS/air system. The thickness of the GUV membrane is not drawn in an actual proportion to improve readability.

As discussed in the main body, when the GUV with a slightly larger diameter than the channel passes through the sensing split, the bottom/top area on the GUV touching split/PDMS channel can be treated approximately as a flatted surface with a thickness of h_{GUV}, i.e., 2.8 nm or 3.5 nm for different phases [14]-[16], as shown in Fig. S6. However, the conformal mapping technique [17] requires both the top and bottom surfaces to cover the whole metal layer. The requirement is not met. Provided that the GUV bottom surface is a thin sheet tightly attached on the metal layer, it can be approximately seen as a dielectric filled in the parallel-plate capacitor formed by the split. Using the conformal mapping in E. Chen *et al*'s work [17], the total capacitance per unit length between two adjacent metals shown in Fig. S6 are written by

$$C_{channel}(\varepsilon_x = \varepsilon_x' - j\varepsilon_x'') = \varepsilon_0 \varepsilon_{air} \frac{K(k_{air}')}{K(k_{air})}\left(1 + \frac{\varepsilon_{sub}-1}{2}\frac{K(k_{sub})}{K(k_{sub}')}\frac{K(k_{air}')}{K(k_{air})} + \frac{\varepsilon_{PDMS}-1}{2}\frac{K(k_{PDMS})}{K(k_{PDMS}')}\frac{K(k_{air}')}{K(k_{air})} + \frac{\varepsilon_{solution}-\varepsilon_{PDMS}}{2}\frac{K(k_{solution})}{K(k_{solution}')}\frac{K(k_{air}')}{K(k_{air})}\right) + \varepsilon_0(\varepsilon_{GUV}-\varepsilon_{solution})\frac{h_{GUV}}{s} \quad (43)$$

and

$$k_{air} = \frac{s}{s+2w} \quad (44)$$

$$k_{sub} = \frac{\sinh(\frac{\pi s}{4h_{sub}})}{\sinh(\frac{\pi(s+2w)}{4h_{sub}})} \tag{45}$$

$$k_{PDMS} = \frac{\sinh(\frac{\pi s}{4h_{PDMS}})}{\sinh(\frac{\pi(s+2w)}{4h_{PDMS}})} \tag{46}$$

$$k_{solution} = \frac{\sinh(\frac{\pi s}{4h_{solution}})}{\sinh(\frac{\pi(s+2w)}{4h_{solution}})} \tag{47}$$

and

$$k_x' = \sqrt{1-k_x^{\ 2}} \tag{48}$$

where *x*=*air*, *sub*, *PDMS*, and *solution*, *K* is incomplete elliptic integral of the first kind with variables k_x and k_x'. It is worth to be mentioned that in Fig. 1(b) the microfluidic channel does not cover the whole metal layer but only ~100 μm long on each side taking the split as the center. So w=100 μm is selected for the calculations of eqs. (43)-(48).

From Fig. 1(b), the microfluidic channel is only 25 μm wide (⊗ direction in Fig. S6), so most of the split is covered by PDMS, not solution. Similarly, the capacitance C_{wall} per unit length for the cross section without the microfluidic channel is expressed as

$$C_{wall}(\varepsilon_x = \varepsilon_x' - j\varepsilon_x'') = \varepsilon_0\varepsilon_{air}\frac{K(k_{air}')}{K(k_{air})}(1+\frac{\varepsilon_{sub}-1}{2}\frac{K(k_{sub})}{K(k_{sub}')}\frac{K(k_{air}')}{K(k_{air})}+\frac{\varepsilon_{PDMS}-1}{2}\frac{K(k_{PDMS})}{K(k_{PDMS}')}\frac{K(k_{air}')}{K(k_{air})}) \tag{49}$$

Consequently, C_{gap} can be obtained as a shunt connection using $C_{channel}$ and C_{wall} in parallel:

$$C_{gap} = w_{channel}C_{channel} + w_{wall}C_{wall} \tag{50}$$

where $w_{channel}$=25 μm is the width of microfluidic channel, and w_{wall}=75 μm is the width of electrodes covered by PDMS.

3 Combinations of Coupled Line and Gaped ML

The *S*-parameters of the symmetric SRR can be solved by analyzing its odd and even modes. Under odd and even mode excitations, it becomes to Fig. S7 (a) and (b) [4]. From

Fig. S7(b), the capacitance C_{gap}, an equivalence of the split, does not play any role under the even-mode excitation.

We can solve the reflections S_{11}^{o} and S_{11}^{e} at Port 1 from Fig. S7 (a) and (b). The complete *S*-parameters of the SRR are expressed as

$$S_{11} = \frac{S_{11}{}^{e} + S_{11}{}^{o}}{2} \tag{51}$$

$$S_{21} = \frac{S_{11}{}^{e} - S_{11}{}^{o}}{2} \tag{52}$$

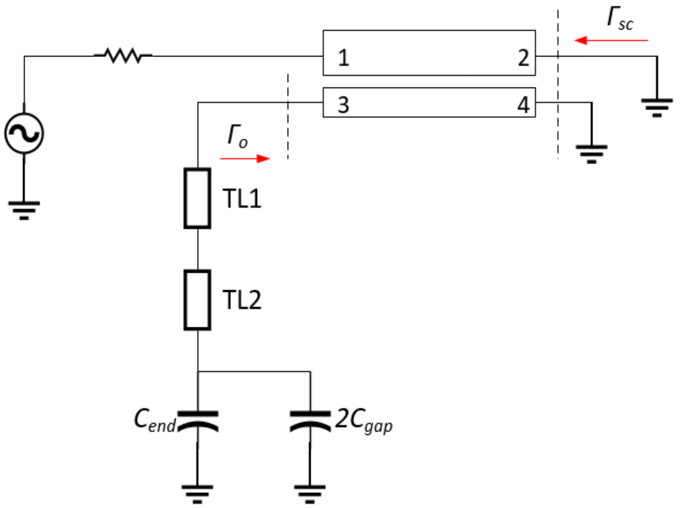

(a)

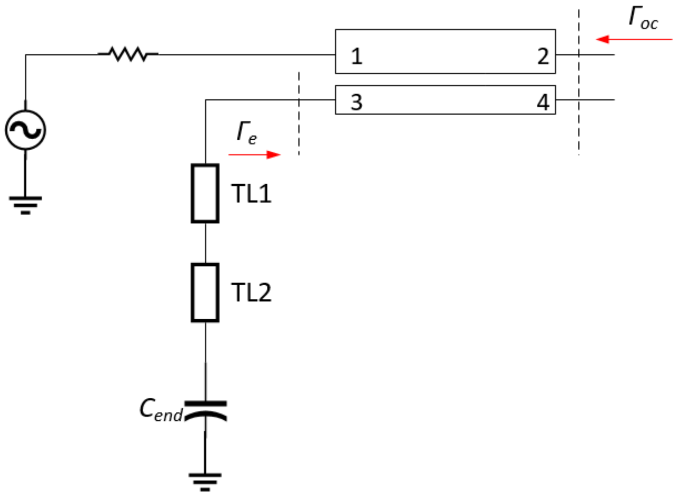

(b)

Fig. S7 Bisection of the circuit of the symmetric SRR under (a) odd-mode and (b) even-mode excitations. The length of the 4-port coupled line needs to be halved here.

The following will take the odd mode as an example to demsonstrate how to solve $S_{11}{}^{o}$. The solving method of $S_{11}{}^{e}$ is similar to $S_{11}{}^{o}$. It is analyzed by the following signal flow [18], where Γ_{sc}=-1 for short-circuit.

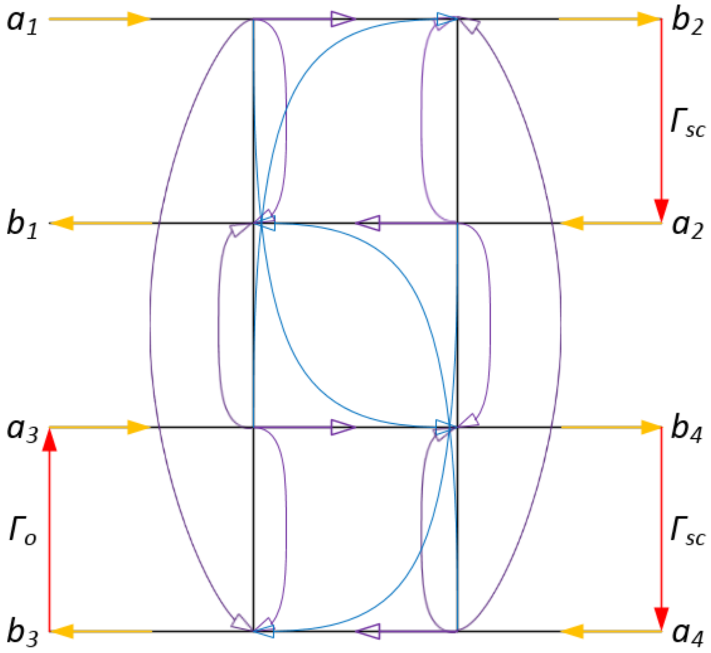

Fig. S8 Signal flow graph for the analysis of $S_{11}{}^{o}$.

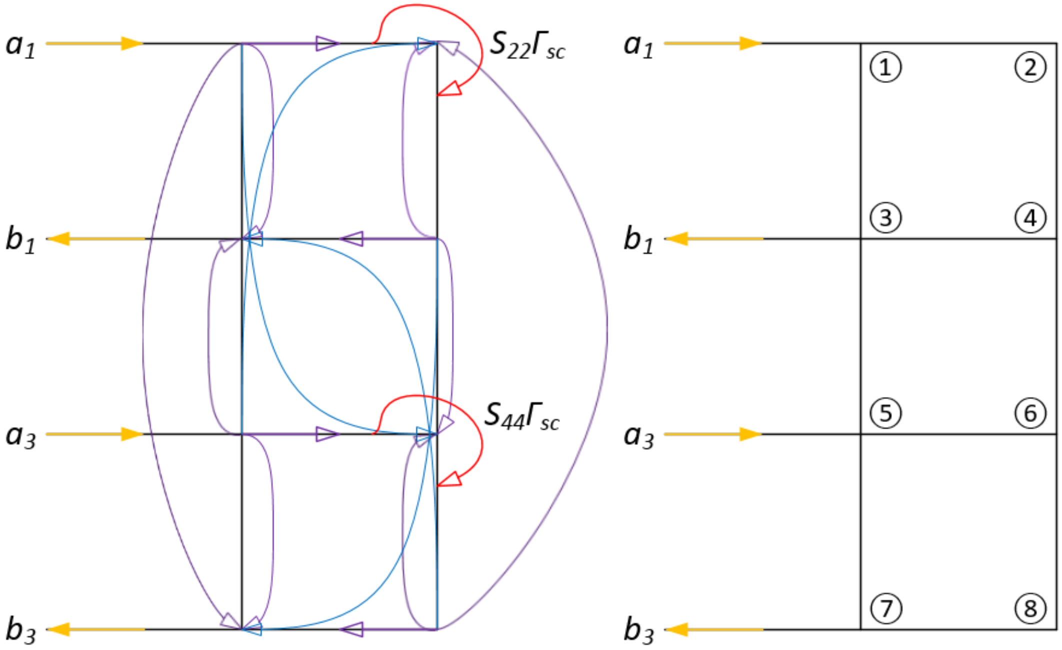

(a) (b)

Fig. S9 (a) Signal flow graph for the analysis of [S_{11}' S_{13}'; S_{31}' S_{33}'] and (b) numbering of the nodes in (a).

To write $S_{11}^{o}=b_1/a_1$ directly is still difficult, so an additional step is necessary, i.e., to match Port 3 (let Γ_o=0) and then solve the 2-port network [S_{11}' S_{13}'; S_{31}' S_{33}'] first, as shown in Fig. S9.

For S_{11}', the signal flow from Node 1 to 3 may be 1→3, 1→2→4→3, and 1→6→8→3. The above three is the simplest. Further, 1243 and 1683 develop 1→2→4→6→8→3, 1→2→4→6→8→2→4→3, 1→2→4→6→8→2→4→6→8→3, 1→2→4→6→8→2→4→6→8→2→4→3, …… and 1→6→8→2→4→3, 1→6→8→2→4→6→8→3, 1→6→8→2→4→6→8→2→4→3, 1→6→8→2→4→6→8→2→4→6→8→3, ……

We can write the expression of S_{11}' using the sum of an infinitely proportional sequence:

$$S_{11}'=S_{11}+\frac{\frac{S_{21}{}^{2}\Gamma_{sc}}{1-S_{22}\Gamma_{sc}}}{1-\frac{S_{42}{}^{2}\Gamma_{sc}{}^{2}}{(1-S_{44}\Gamma_{sc})(1-S_{22}\Gamma_{sc})}}+\frac{\frac{S_{21}S_{42}S_{14}\Gamma_{sc}{}^{2}}{(1-S_{22}\Gamma_{sc})(1-S_{44}\Gamma_{sc})}}{1-\frac{S_{42}{}^{2}\Gamma_{sc}{}^{2}}{(1-S_{22}\Gamma_{sc})(1-S_{44}\Gamma_{sc})}}$$
$$+\frac{\frac{S_{41}{}^{2}\Gamma_{sc}}{1-S_{44}\Gamma_{sc}}}{1-\frac{S_{24}{}^{2}\Gamma_{sc}{}^{2}}{(1-S_{22}\Gamma_{sc})(1-S_{44}\Gamma_{sc})}}+\frac{\frac{S_{41}S_{24}S_{12}\Gamma_{sc}{}^{2}}{(1-S_{44}\Gamma_{sc})(1-S_{22}\Gamma_{sc})}}{1-\frac{S_{42}{}^{2}\Gamma_{sc}{}^{2}}{(1-S_{44}\Gamma_{sc})(1-S_{22}\Gamma_{sc})}} \quad (53)$$

For S_{33}', the signal flow from Node 5 to 7 may be 5→7, 5→2→4→7, 5→2→4→6→8→7, 5→2→4→6→8→2→4→7, 5→2→4→6→8→2→4→6→8→7, 5→2→4→6→8→2→4→6→8→2→4→7, …… and 5→6→8→7, 5→6→8→2→4→7, 5→6→8→2→4→6→8→7, 5→6→8→2→4→6→8→2→4→7, 5→6→8→2→4→6→8→2→4→6→8→7, …… Similarly, S_{33}' is in the form of

$$S_{33}' = S_{33} + \frac{\frac{S_{23}^{2}\Gamma_{sc}}{1-S_{22}\Gamma_{sc}}}{1-\frac{S_{42}^{2}\Gamma_{sc}^{2}}{(1-S_{44}\Gamma_{sc})(1-S_{22}\Gamma_{sc})}} + \frac{\frac{S_{23}S_{42}S_{34}\Gamma_{sc}^{2}}{(1-S_{22}\Gamma_{sc})(1-S_{44}\Gamma_{sc})}}{1-\frac{S_{42}^{2}\Gamma_{sc}^{2}}{(1-S_{22}\Gamma_{sc})(1-S_{44}\Gamma_{sc})}}$$
$$+\frac{\frac{S_{43}^{2}\Gamma_{sc}}{1-S_{44}\Gamma_{sc}}}{1-\frac{S_{24}^{2}\Gamma_{sc}^{2}}{(1-S_{22}\Gamma_{sc})(1-S_{44}\Gamma_{sc})}} + \frac{\frac{S_{34}S_{24}S_{23}\Gamma_{sc}^{2}}{(1-S_{44}\Gamma_{sc})(1-S_{22}\Gamma_{sc})}}{1-\frac{S_{42}^{2}\Gamma_{sc}^{2}}{(1-S_{44}\Gamma_{sc})(1-S_{22}\Gamma_{sc})}} \tag{54}$$

For S_{31}', the signal flow from Node 1 to 7 may be 1→7, 1→2→4→7, 1→2→4→6→8→7, 1→2→4→6→8→2→4→7, 1→2→4→6→8→2→4→6→8→7, 1→2→4→6→8→2→4→6→8→2→4→7, …… and 1→6→8→7, 1→6→8→2→4→7, 1→6→8→2→4→6→8→7, 1→6→8→2→4→6→8→2→4→7, 1→6→8→2→4→6→8→2→4→6→8→7, …… Similarly, S_{31}' is in the form of

$$S_{31}' = S_{13}' = S_{31} + \frac{\frac{S_{21}S_{32}\Gamma_{sc}}{1-S_{22}\Gamma_{sc}}}{1-\frac{S_{42}^{2}\Gamma_{sc}^{2}}{(1-S_{44}\Gamma_{sc})(1-S_{22}\Gamma_{sc})}} + \frac{\frac{S_{21}S_{42}S_{34}\Gamma_{sc}^{2}}{(1-S_{22}\Gamma_{sc})(1-S_{44}\Gamma_{sc})}}{1-\frac{S_{42}^{2}\Gamma_{sc}^{2}}{(1-S_{22}\Gamma_{sc})(1-S_{44}\Gamma_{sc})}}$$
$$+\frac{\frac{S_{41}S_{34}\Gamma_{sc}}{1-S_{44}\Gamma_{sc}}}{1-\frac{S_{24}^{2}\Gamma_{sc}^{2}}{(1-S_{22}\Gamma_{sc})(1-S_{44}\Gamma_{sc})}} + \frac{\frac{S_{41}S_{24}S_{32}\Gamma_{sc}^{2}}{(1-S_{44}\Gamma_{sc})(1-S_{22}\Gamma_{sc})}}{1-\frac{S_{42}^{2}\Gamma_{sc}^{2}}{(1-S_{44}\Gamma_{sc})(1-S_{22}\Gamma_{sc})}} \tag{55}$$

Now, we can draw the signal flow of the 2-port network [S_{11}' S_{13}'; S_{31}' S_{33}'] to solve S_{11}^{o}. It is obtained by eq. (56). Using the similar steps, S_{11}^{e} can be also solved.

$$S_{11}^{o} = S_{11}' + \frac{S_{31}'S_{13}'\Gamma_{o}}{1-S_{33}'\Gamma_{o}} \tag{56}$$

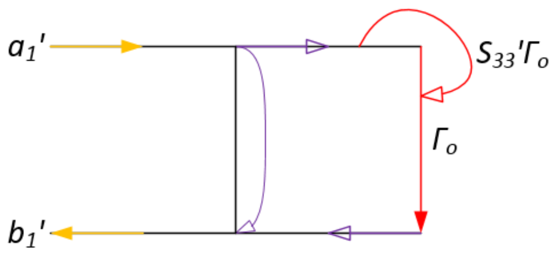

Fig. S10 Signal flow graph of the network [S_{11}' S_{13}'; S_{31}' S_{33}'].

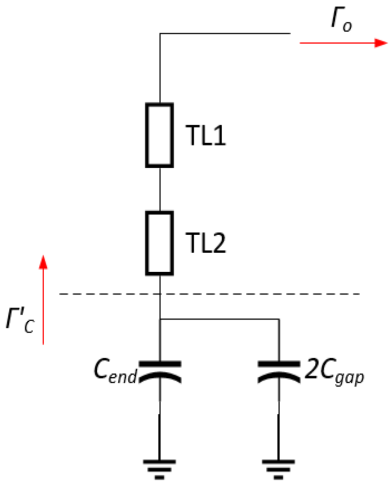

Fig. S11 1-port network decomposed from Fig. S7(a) for Γ_o.

The rest of the work is to solve the reflection coefficient Γ_o and Γ_e in Fig. S7 (a) and (b). Taking Γ_o for example, it is S_{11} of the 1-port network in Fig. S11, which is decomposed from Fig. S7(a). The 2-port S-parameters [S_{11}" S_{12}"; S_{21}" S_{22}"] of the transmission line TL1 and TL2 in series in Fig. S11 can be easily solved. The calculations of conductor loss and 45° mitered bend can be found in [19], [20]. Then, S_{11} of the network, i.e., Γ_o, is expressed as eq. (57). The similar process is also used to obtain Γ_e.

$$\Gamma_o = S_{11}" + \frac{S_{21}"S_{12}"\Gamma'_C}{1 - S_{22}"\Gamma'_C} \tag{57}$$

where the reflection coefficient Γ_C' is

$$\Gamma_C' = \frac{1 - Z_0 j\omega(C_{end} + 2C_{gap})}{1 - Z_0 j\omega(C_{end} + 2C_{gap})} \quad . \tag{58}$$

For the SRR loading 0.1 M glucose-water solution in Fig. 1(b), the calculated $|S_{21}|$ from 1 GHz to 12 GHz using the proposed analytical model is shown in Fig. S12. The frequency-dependent complex permittivity of the solution [21] is used to calculate eq. (43). To analyze the respective resonant frequencies of odd-mode and even-mode, $|S_{11}^{o}|$ for odd-mode and $|S_{11}^{e}|$ for even-mode are also demonstrated, respectively, in Fig. S13 (a) and (b). We can see that 1st and 2nd resonant frequencies of the odd-mode, i.e., 2.68 GHz and 7.99 GHz, correspond to 1st and 3rd resonant frequencies of the complete SRR, i.e., 2.54 GHz and 7.54 GHz as shown in Fig. S12, whereas 1st and 2nd resonant frequencies of the even-mode, i.e., 5.08 GHz and 10.99 GHz, correspond to 2nd and 4th resonant frequencies of the

complete SRR, i.e., 4.77 GHz and 9.90 GHz. The deviations are caused since only half of full length is calculated for $|S_{11}^{o}|$ and $|S_{11}^{e}|$, which is a requirement of the even-odd mode analysis technique [4]. It is also observed that the values at these frequencies for the combined $|S_{21}|$ are different from the single $|S_{11}^{o}|$ and $|S_{11}^{e}|$, due to the combination calculation in eq. (52). The dimension (not included in Fig. 1c) of the proposed SRR in the calculation is shown in Fig. S14.

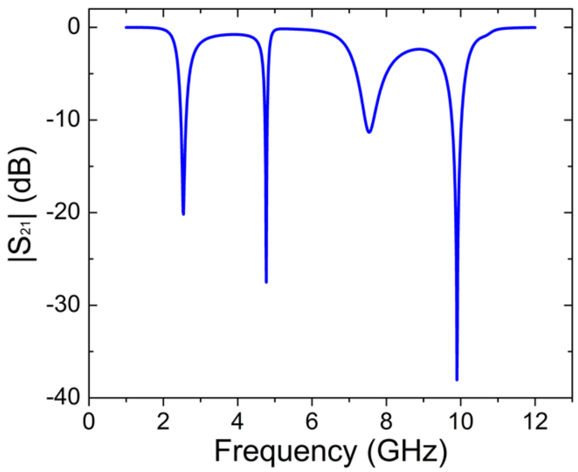

Fig. S12 Calculated $|S_{21}|$ from 1 GHz to 12 GHz loading 0.1 M glucose-water solution.

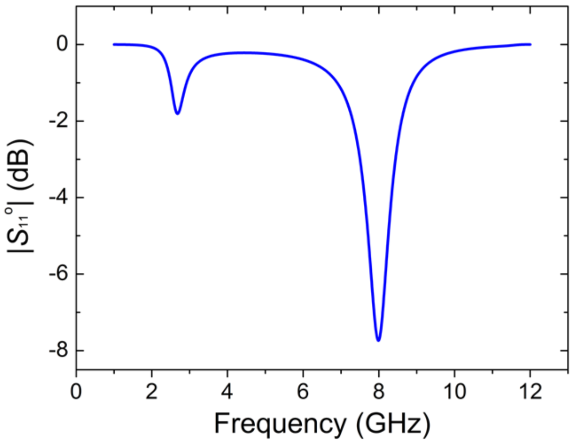

(a)

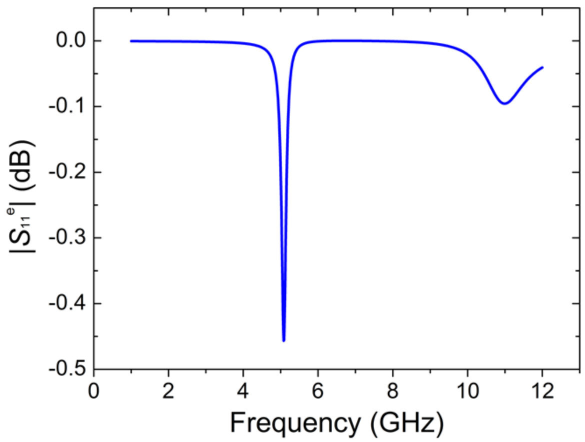

(b)

Fig. S13 (a) $|S_{11}^{o}|$ for the odd-mode and (b) $|S_{11}^{e}|$ for the even-mode.

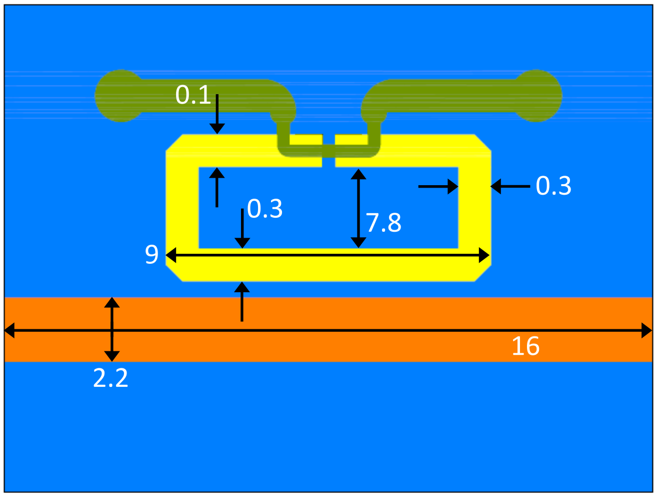

Fig. S14 Dimension of the proposed SRR (Unit: mm).

4 Complex Permittivity Extraction of GUV Membrane

In Fig. S12, 1st frequency locates at 2.54 GHz with a $|S_{21}|$ of ~-20.17 dB, much lower than the measured ~-4.51 dB in Fig. 1(d), implying that the proposed coupled-line model has a stronger coupling between two adjacent MLs. The same thing also affects other resonant frequencies. The reason is that the calculation of the mutual-capacitance coefficients c_{31} for two adjacent asymmetric lines uses the coplanar stripline (CPS)-based conformal mapping [7], but it is unlike two coplanar MLs of the SRR. For the CPS, almost all electric field streamlines emitted from an electrode terminate at another one; for two adjacent MLs of the SRR, a lot of electric field streamlines emitted from an electrode terminate at the back conductor [7]. So the calculated c_{31} is larger than what it should be. Moreover, the proposed model is derived based on the assumption of TEM propagation, which has to be used to meet the prerequisite of eq. (6.2), but it is only strictly satisfied in a homogeneous medium. Based on this assumption, the field distribution at any transverse plane perpendicular to z-direction in Fig. S2 can be treated as a linear combination of the proposed two fundamental TEM-modes, i.e., π Mode and c Mode in Section 1. Then the derivations in Section 1 can be further performed for a relatively simple and intuitive analysis of the SRR.

To extract the permittivity of the measured GUV membranes using the proposed model, some additional revisions are required. According to the above discussion, c_{31} is the

main target. For s=3.5 μm (the spacing between two adjacent MLs), c_{31} is calculated to be ~95.60 pF, demonstrating a strong coupling in Fig. S12. If it is decreased to ~19.11 pF, the $|S_{21}|$ at 1st resonant frequency is close to the measured value, i.e., ~-4.51 dB. The minor frequency error is eliminated by decreasing the length of the coupled line. Then the corrected $|S_{21}|$ from 1 GHz to 12 GHz is re-drawn in Fig. S15(a). But, 2nd resonant frequency shifts to 8.1 GHz with a $|S_{21}|$ of -1.53 dB, therefore. So the above adjustment is more suitable for permittivity extraction at 2.7 GHz, and c_{31} and the length of the coupled line need to be adjusted again when extracting permittivity at 7.9 GHz. Figure S15(b) also demonstrates the corrected $|S_{21}|$ from 1 GHz to 12 GHz using a c_{31} of ~28.90 pF, which agrees with the measured $|S_{21}|$ at 7.9 GHz.

To verify the proposed model, it is necessary to measure another MUT with known permittivity and compare it with the calculated result. Replacing 0.1 M water-glucose solution with air (ε_{air}≈1-j0), the calculated $|S_{21}|$s using 19.11 pF and 28.90 pF as c_{31}, respectively, for good agreement at 2.7 GHz and 7.9 GHz, are compared with the measured data together in Table S1. The relative errors at 2.7 GHz and 7.9 GHz from Table S1 are 1.00% and 1.90%, respectively, suggesting that the proposed model can obtain an accurate permittivity of MUT. It is worth noting that the above comparison is made for a wide permittivity range (from the solution to the air). In fact, for the minute permittivity change without and with a GUV membrane, the proposed model is expected to obtain a lower relative error.

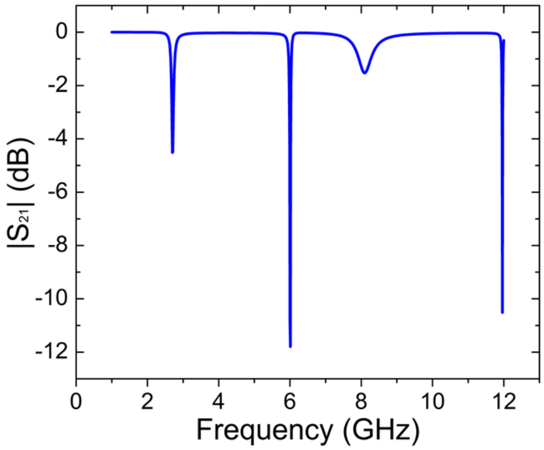

(a)

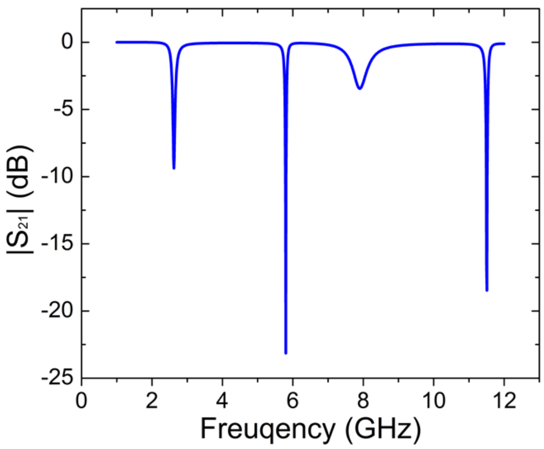

(b)

Fig. S15 Corrected $|S_{21}|$ from 1 GHz to 12 GHz loading 0.1 M glucose-water solution demonstrating a good agreement with the measurement at (a) 2.7 GHz and at (b) 7.9 GHz.

TABLE S1
COMPARISON BETWEEN THE CALCULATED AND THE MEASURED $|S_{21}|$S USING AIR AS A MUT AT 2.7 GHZ AND 7.9 GHZ

Frequency	Calculated $\|S_{21}\|$	Measured $\|S_{21}\|$	Relative error*
2.7 GHz	-0.02526 dB	0.06129 dB#	1.00%
7.9 GHz	-0.04479 dB	-0.2079 dB	1.90%

*Relative error=|(Calculated-Measured)/Measured|×100%, where the data is in linear format, rather than dB, for the calculation. #The value is slightly larger than 0 dB, which is caused by the calibration error of VNA.

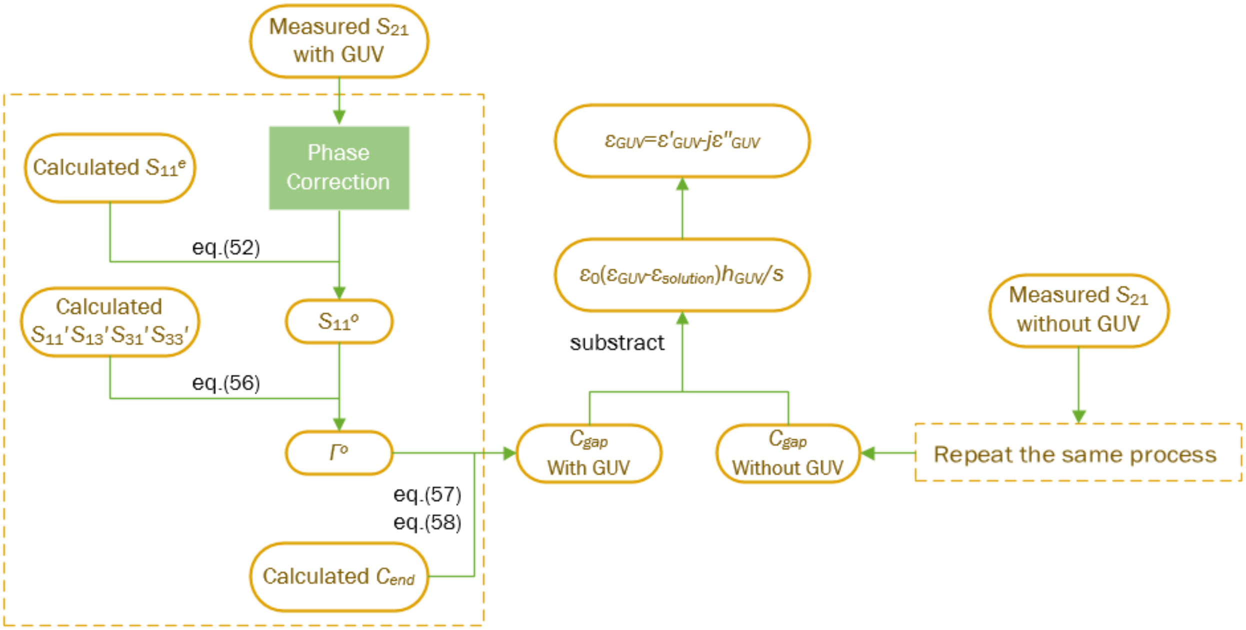

Fig. S16 Complete calculation flow graph for GUV membrane complex permittivity.

Until this step, however, the additional phase shift induced by the left and right MLs in Fig. S1 is not taken into account. In Fig. S15, the S_{21} at 1st resonant frequency 2.7 GHz is 315.89°, which should be corrected to the measured angle, i.e., 104.01°. Then the data at 2.7 GHz in Fig. 3 can be transformed to corresponding complex permittivities in Fig. 8. The same procedures are also done at 7.9 GHz. In the transformation, the width of the flattened GUV membrane (along W direction in Fig. S6) w_{GUV} is selected as 25 μm. But, according to

the discussion in 5th paragraph of the main body, the possible w_{GUV} value ranges from 20.8 μm to 25 μm. For other possible choices of w_{GUV}, the extracted complex permittivities may demonstrate a slight deviation. Figure S16 shows a complete calculation flow graph for GUV membrane complex permittivity using the proposed model.

References

[1] J. D. Baena, J. Bonache, F. Martín, R. M. Sillero, F. Falcone, T. Lopetegi, M. A. G. Laso, J. García–García, I. Gil, M. F. Portillo, and M. Sorolla, "Equivalent-circuit models for split-ring resonators and complementary split-ring resonators coupled to planar transmission lines," *IEEE Trans. Microw. Theory Techn.*, vol. 53, no. 4, pp. 1451-1461, Apr. 2005.

[2] H. –J. Lee, H. –S. Lee, K. –H. Yoo, and J. –G. Yook, "DNA sensing using split-ring resonator alone at microwave regime," *Appl. Phys. Lett.*, vol. 108, no. 1, pp. 014908, Jul. 2010.

[3] C. –S. Lee and C. –L. Yang, "Single-compound complementary split-ring resonator for simultaneously measuring the permittivity and thickness of dual-layer dielectric materials," *IEEE Trans. Microw. Theory Techn.*, vol. 63, no. 6, pp. 2010-2023, Jun. 2015.

[4] J. Reed and G. J. Wheeler, "A Method of Analysis of Symmetrical Four-Port Networks," *IRE Trans. Microwave Theory Tech.*, vol. MTT-4, pp. 246-252, Oct. 1956.

[5] D. M. Pozar, *Microwave Engineering*, 4th ed. New York: Wiley, 2012, pp. 175-176.

[6] D. Pavlidis and H. L. Hartnagel, "The design and performance of three-line microstrip couplers," *IEEE Trans. Microw. Theory Techn.*, vol. MTT-24, no. 10, pp. 631-640, Oct. 1976.

[7] R. Garg and I. J. Bahl, "Characteristics of coupled microstriplines," *IEEE Trans. Microw. Theory Techn.*, vol. MTT-27, no. 7, pp. 700-705, Jul. 1979.

[8] G. Ghione, "A CAD-oriented analytical model for the losses of general asymmetric coplanar lines in hybrid and monolithic MICs," *IEEE Trans. Microwave Theory Tech.*, vol. 41, no. 9, pp. 1499-1510, Sep. 1993.

[9] V. K. Tripathi, and Y. K. Chin, "Analysis of the general nonsymmetrical directional coupler with arbitrary terminations," *Proc. Inst. Elect. Eng. —Microw., Antennas, Propag.*, vol. 129, no. 6, pt. H, pp. 360-362, Dec. 1982.

[10] M. Meada, "An analysis of gap in microstrip transmission lines," *IEEE Trans. Microw. Theory Techn.*, vol. MTT-20, no. 6, pp. 390-396, Jun. 1972.

[11] A. Farrar and A. T. Adams, "Matrix methods for microstrip three-dimensional problems," *IEEE Trans. Microw. Theory Techn.*, vol. MTT-20, no. 8, pp. 497-504, Aug. 1972.

[12] P. Benedek and P. Silvester, "Equivalent capacitances for microstrip gaps and steps," *IEEE Trans. Microw. Theory Techn.*, vol. MTT-20, no. 11, pp. 729-733, Nov. 1972.

[13] J. –S. Hong and M. J. Lancaster, *Microstrip Filters for RF/Microwave Applications*, 1st ed. New York: Wiley, 2001, pp. 89-92.

[14] A. Pralle, P. Keller, E. -L. Florin, K. Simons, and J. Hörber, "Sphingolipid-cholesterol rafts diffuse as small entities in the plasma membrane of mammalian cells," *J. Cell Biol.*, vol. 148, no. 5, pp. 997-1008, Mar. 2000.

[15] K. Simons, and E. Ikonen, "Functional rafts in cell membranes," *Nature*, vol. 387, pp. 569-572, Jun. 1997.

[16] W. H. Binder, V. Barragan, and F. M. Menger, "Domains and rafts in lipid membranes," *Angew. Chem. Int. Ed.*, vol. 42, 5802-5827, Dec. 2003.

[17] E. Chen and S. Y. Chou, "Characteristics of coplanar transmission lines on multilayer substrates: modeling and experiments," *IEEE Trans. Microwave Theory Tech.*, vol. 45, no. 6, pp. 939-945, June 1997.

[18] D. M. Pozar, *Microwave Engineering*, 4th ed. New York: Wiley, 2012, pp. 194-197.

[19] R. A. Pucel, D. J. Masse, and C. P. Hartwig, "Losses in microstrip," *IEEE Trans. Microwave Theory Tech.*, vol. MTT-16, vol. 16, no. 6, pp. 342-350, June 1968.

[20] R. Kirschning, H. Jansen, and N. H. L. Koster, "Measurement and computer-aided modeling of microstrip discontinuities by an improved resonator method," *IEEE MTT-S Int. Microw. Symp.*, pp. 495-497, May 1983.

[21] H. Weingärtner, A. Knocks, S. Boresch, P. Höchtl, and O. Steinhauser, "Dielectric spectroscopy in aqueous solutions of oligosaccharides: experiment meets simulation," *J. Chem. Phys.*, vol. 115, no. 3, pp. 1463-1472, Jul. 2001.
